# Predictors of willingness to participate in COVID-19 clinical trials among Black and Latino adults

**DOI:** 10.1017/cts.2024.654

**Published:** 2024-11-22

**Authors:** Christine M. Weston, Elizabeth L. Andrade, Wuraola Olawole, Monica Guerrero Vazquez, Hailey Miller, Sarah C. Stevens, Cyd Lacanienta, Nancy Perrin, Mark C. Edberg, Thomas A. Mellman, Yvonne Bronner, Roger Clark, Cheryl R. Dennison Himmelfarb

**Affiliations:** 1 Johns Hopkins Bloomberg School of Public Health, Baltimore, MD, USA; 2 George Washington University Milken Institute School of Public Health, Washingon, DC, USA; 3 Johns Hopkins University School of Nursing, Baltimore, MD, USA; 4 Johns Hopkins University Centro SOL, Baltimore, MD, USA; 5 Johns Hopkins University School of Medicine, Baltimore, MD, USA; 6 Johns Hopkins University Institute for Clinical and Translational Research, Baltimore, MD, USA; 7 Howard University, Washington, DC, USA; 8 Morgan State University, Baltimore, MD, USA

**Keywords:** COVID-19, clinical trial participation, African Americans/Blacks, Latinos, social determinants of health

## Abstract

**Introduction:**

Black and Latino individuals are underrepresented in COVID-19 treatment and vaccine clinical trials, calling for an examination of factors that may predict willingness to participate in trials.

**Methods:**

We administered the Common Survey 2.0 developed by the Community Engagement Alliance (CEAL) Against COVID-19 Disparities to 600 Black and Latino adults in Baltimore City, Prince George’s County, Maryland, Montgomery County, Maryland, and Washington, DC, between October and December 2021. We examined the relationship between awareness of clinical trials, social determinants of health challenges, trust in COVID-19 clinical trial information sources, and willingness to participate in COVID-19 treatment and vaccine trials using multinomial regression analysis.

**Results:**

Approximately half of Black and Latino respondents were unwilling to participate in COVID-19 treatment or vaccine clinical trials. Results showed that increased trust in COVID-19 clinical trial information sources and trial awareness were associated with greater willingness to participate in COVID-19 treatment and vaccine trials among Black and Latino individuals. For Latino respondents, having recently experienced more challenges related to social determinants of health was associated with a decreased likelihood of willingness to participate in COVID-19 vaccine trials.

**Conclusions:**

The willingness of Black and Latino adults to participate in COVID-19 treatment and vaccine clinical trials is influenced by trial awareness and trust in trial information sources. Ensuring the inclusion of these communities in clinical trials will require approaches that build greater awareness and trust.

## Introduction

The underrepresentation of historically marginalized communities in clinical research is a long-standing challenge that threatens health outcomes and compounds health disparities [1]. The COVID-19 pandemic highlighted profound disparities in healthcare access, treatment [2–3], and outcomes [4–10] among historically marginalized groups, which led to renewed attention to the underrepresentation of Black and Latino communities in clinical trials and a sense of urgency to understand and develop effective solutions to promote diverse and inclusive participation in clinical research [11].

Evidence of the underrepresentation of minoritized communities in clinical research continues to mount. A 2023 study of 9,869 patients found that Latino, American Indian/Alaska Native, and Black participants were significantly underrepresented, and White participants were significantly overrepresented in lung cancer clinical trials. Even more remarkable, this study discovered that disparities for Latino patients worsened from 2017 to 2021 and that unequal representation of these racial and ethnic groups in clinical trials has not improved since 2004 [12].

Reports have also indicated that Black and Latino individuals were less willing to participate in COVID-19 treatment and vaccine clinical trials compared with White individuals [13–16]. A recent 2023 study of 14,397 adults from the American Heart Association’s COVID-19 Cardiovascular Registry found that Black patients hospitalized with COVID-19 had the lowest enrollment in clinical trials (8%) compared to all other racial groups [17].

A recent systematic review of 122 US-based COVID-19 prevention and treatment clinical trials comprising 176,654 participants found that Black participants were underrepresented in treatment trials, signaling potential barriers, and mistrust in biomedical research [18]. The same study found that Latino participants were overrepresented in treatment trials, in contrast to prior studies, and likely reflective of lower access to primary care services and increased risk of hospitalization. Therefore, understanding attitudes, perceptions, and barriers to clinical trial participation is fundamental to developing successful interventions to increase diverse representation in clinical trials.

Recognition of the disproportionate effect of COVID-19 on communities of color prompted the National Institutes of Health (NIH) to fund the Community Engagement Alliance (CEAL) Against COVID-19 Disparities to provide education, support, and resources to the communities hit hardest by the COVID-19 pandemic. One of the primary goals of CEAL was to develop, implement, and test collaborative community engagement strategies to improve the uptake of vaccines and increase participation in COVID-19 vaccine and treatment clinical trials in vulnerable communities. The current study examines the relationship between demographics, social determinants of health challenges, awareness of clinical trials, and trust in COVID-19 clinical trial information sources with willingness to participate in COVID-19 treatment and vaccine trials among non-Latino Black and Latino individuals residing in the greater Baltimore and Washington, DC metropolitan areas. While various studies have examined Black and Latino willingness and barriers to participation in clinical research, few, if any, have examined the relationship between clinical trials awareness, trust in information about clinical trials, and social determinants of health as predictors of willingness to participate in clinical trials, and even fewer have focused on COVID-19 treatment and vaccine trials in particular.

## Material and methods

We administered a cross-sectional survey from October to December 2021 in two geographic areas: 1) Baltimore, Maryland, conducted by Johns Hopkins University (JHU), and 2) Prince George’s and Montgomery Counties, Maryland, conducted by the George Washington University (GW). Both universities worked in close collaboration with their community partners for survey administration. All study activities were approved by the Johns Hopkins Medicine Institutional Review Boards (#IRB00299468).

The survey instrument, the Common Survey 2.0, was designed by the NIH CEAL network and contained questions in the following domains: demographics; healthcare utilization; social determinants of health challenges; COVID-19 information trust and risk perceptions; COVID-19 prevention, testing, and vaccination; and research participation. It had an overall Flesch–Kincaid reading level of 6.9 and was available in English and Spanish. All participants were required to be 18 years of age or older and a resident of the Baltimore or Washington, DC, greater metropolitan areas.

We recruited participants in Baltimore City using convenience sampling at public markets, community events, and food distribution sites in areas with low COVID-19 vaccination rates, as reported by the Baltimore City COVID-19 Response Taskforce. We had a recruitment flyer in Spanish and English, but most participants were approached in-person at the recruitment locations and verbally invited to participate (in both Spanish and English). For the Baltimore Latino population specifically, we used social media, as well as existing connections with community members through Centro Sol, a community center providing advocacy, outreach, education, opportunities, and youth programs for the Baltimore Latino Population. We recruited participants from Prince George’s County, Maryland, Montgomery County, Maryland, and Washington, DC, using convenience sampling from three churches with high COVID-19 transmission and low vaccination rates near Langley Park, Maryland. For the GW sample, survey respondents (all Latino and Spanish-speaking) were recruited through one of three ways: 1) through social media platforms within the networks of team community health workers (CHWs) and our partner organizations social media pages (a digital flyer was used); 2) through announcements on a daily health-focused radio talk show in Spanish, *Consultorio Comunitario,* on AM Radio America; and 3) through local partner churches (print flyer and announcements were used).

All data collectors completed CITI training for the protection of human subjects and received additional training on how to administer the survey. JHU had six English-speaking and three Spanish-speaking interviewers, and GW had 14 Spanish-speaking interviewers. Interviewers recorded participants’ responses directly into REDCap using password-protected, internet-connected tablets. The survey took approximately 25 minutes, and participants were compensated $25 for their time.

### Measures

**Social determinants of health challenges** assessed the mean severity of challenges respondents faced in the past month. The subscale consisted of four items, including “Having a place to live,” “Getting enough food to eat,” “Getting the medications I need,” and “Getting where I need to go.” The response scale ranged from 1, “No, this is not a challenge,” to 3, “Yes, this is a major challenge,” and had a Cronbach’s alpha of 0.85. We computed the mean scale score across all items (provided at least 75% of items were present).

**Trust in COVID-19 clinical trial information sources** measured the mean level of trust in COVID-19 clinical trial information sources. The measure consisted of seven items, including the “National Institutes of Health;” “your doctor or healthcare provider;” “your local healthcare clinic or hospital;” “university hospitals;” “companies that make drugs for medical use;” “people who do research,” and “friends, family, and community leaders.” The response scale ranged from 1, “Not at all,” to 3, “A lot,” and had a Cronbach’s alpha of 0.90. We computed the mean scale score across all items (provided at least 75% of items were present).

**Awareness of COVID-19 clinical trials** was a binary variable assessing respondents’ awareness of COVID-19 clinical trials for treatments or vaccines (No or Yes).

### Outcome variables

The two outcome variables were 1) willingness to sign up for a COVID-19 treatment clinical trial and 2) willingness to sign up for a COVID-19 vaccine clinical trial assessed on a scale from 1, “Not willing” to 7, “Very willing.” The distribution for both outcomes was bimodal, such that most participants were either “not willing” or “very willing” to participate, leading to small counts in intermediate response groups. Hence, outcome variables were collapsed into three groups to maximize power: 1 and 2, “Not willing;” 3, 4, and 5, “Somewhat willing;” and 6 and 7, “Very willing.”

### Statistical analysis

We used multinomial regression to examine the factors that predicted willingness to sign up for COVID-19 clinical trials using two models: one for willingness to participate in COVID-19 treatment trials and one for willingness to participate in COVID-19 vaccine trials. The predictor variables were 1) social determinants of health challenges, 2) trust in COVID-19 trial information sources, and 3) awareness of COVID-19 clinical trials. Both unadjusted and adjusted models were estimated. Adjusted models included all predictors from the unadjusted models and demographic characteristics that were significantly associated with willingness to participate in clinical trials. In exploratory analyses, we examined if the predictors of willingness to participate differed between Black and Latino respondents by including interaction terms for race/ethnicity in the unadjusted models. Significant interactions were followed by stratified models to facilitate the interpretation of results. We used R version 4.2.2 for all analyses and nnet (version 7.3-18) R package to build the multinomial models. We used *p* < 0.05 to determine statistical significance.

## Results

Six hundred non-Latino Black (38.3%) and Latino (61.7%) participants completed the survey. Overall, 63% were female, 67% had a high school education or less, 31% had an annual household income of $15,000 or less (31%), and the average age was 44.8 years. The differences in characteristics between Black and Latino participants are shown in Table 1.

**Table 1.** Sample characteristics among Black and Latino participants

	Total	Black non-Latino, *N* = 230	Latino, *N* = 370	P-value
Survey language				< 0.001
English	237.0 (39.5%)	230.0 (100.0%)	7.0 (1.9%)	
Spanish	363.0 (60.5%)	0.0 (0.0%)	363.0 (98.1%)	
Gender				0.021
Man	218.0 (36.6%)	97.0 (42.4%)	121.0 (33.0%)	
Woman	378.0 (63.4%)	132.0 (57.6%)	246.0 (67.0%)	
Age	44.8 (14.8)	50.4 (15.0)	41.2 (13.6)	< 0.001
Education				< 0.001
Less than or some high school	171.0 (29.2%)	45.0 (19.6%)	126.0 (35.4%)	
High school/GED	224.0 (38.2%)	119.0 (51.7%)	105.0 (29.5%)	
Some college or associate degree	125.0 (21.3%)	46.0 (20.0%)	79.0 (22.2%)	
Bachelor’s degree or more	66.0 (11.3%)	20.0 (8.7%)	46.0 (12.9%)	
Income				0.078
Less than $15,000	162.0 (31.2%)	67.0 (32.4%)	95.0 (30.4%)	
$15,000-$34,999	183.0 (35.3%)	82.0 (39.6%)	101.0 (32.4%)	
$35,000+	174.0 (33.5%)	58.0 (28.0%)	116.0 (37.2%)	
Employment status				
Part-time	134.0 (22.6%)	18.0 (7.8%)	116.0 (32.0%)	< 0.001
Full time	220.0 (37.1%)	75.0 (32.6%)	145.0 (39.9%)	0.072
Retired from work	53.0 (8.9%)	32.0 (13.9%)	21.0 (5.8%)	< 0.001
Not able to work due to disability	49.0 (8.3%)	40.0 (17.4%)	9.0 (2.5%)	< 0.001
Unemployed	103.0 (17.4%)	68.0 (29.6%)	35.0 (9.6%)	< 0.001
Other	45.0 (7.6%)	9.0 (3.9%)	36.0 (9.9%)	0.007
Have a health insurance plan				< 0.001
Yes	431.0 (72.9%)	219.0 (95.6%)	212.0 (58.6%)	
No	160.0 (27.1%)	10.0 (4.4%)	150.0 (41.4%)	

### Social determinants of health challenges, trust in clinical trial information sources, and awareness of COVID-19 clinical trials

Participants experienced moderate social determinants of health challenges with a mean score of 1.41 on a scale of 1 to 3. The mean trust in COVID-19 clinical trial information sources was relatively high (*M* = 2.45). On average, 21% of participants indicated that they were aware of COVID-19 clinical trials that were being done (Table 2).

**Table 2.** Predictor and outcome variables among Black and Latino participants

	Total	Black non-Latino, *N* = 230	Latino, *N* = 370	P-value
Social determinants of health challenges subscale	1.41 (0.56)	1.35 (0.55)	1.46 (0.56)	0.002
Trust in doctor subscale	3.66 (1.00)	3.71 (0.95)	3.62 (1.04)	0.301
Trust in COVID-19 clinical trial info sources subscale	2.45 (0.53)	2.51 (0.60)	2.41 (0.48)	< 0.001
COVID-19 clinical trial awareness	115.0 (20.5%)	42.0 (18.5%)	73.0 (21.8%)	0.343
Willingness to sign up for a COVID-19 treatment trial				0.772
Not willing	309.0 (52.6%)	124.0 (54.4%)	185.0 (51.5%)	
Somewhat willing	123.0 (21.0%)	45.0 (19.7%)	78.0 (21.7%)	
Very willing	155.0 (26.4%)	59.0 (25.9%)	96.0 (26.7%)	
Willingness to sign up for COVID-19 trial				0.264
Not willing	332.0 (56.9%)	123.0 (53.9%)	209.0 (58.9%)	
Somewhat willing	130.0 (22.3%)	50.0 (21.9%)	80.0 (22.5%)	
Very willing	121.0 (20.8%)	55.0 (24.1%)	66.0 (18.6%)	

### Willingness to participate in a COVID-19 treatment trial

Approximately half of the participants (52.6%) were unwilling to participate in a COVID-19 treatment trial, 21.0% were somewhat willing, and 26.4% were very willing. Willingness to participate in treatment trials did not differ between Black and Latino respondents (Table 2).

### Willingness toparticipate in COVID-19 vaccine trial

Approximately half of the participants (56.9%) were unwilling to participate in a COVID-19 vaccine trial, 22.3% were somewhat willing, and 20.8% were very willing. Willingness to participate in vaccine trials did not differ between Black and Latino respondents (Table 2).

### Multinomial regression of predictors for willingness to sign up for a COVID-19 treatment trial or vaccine trial

In the adjusted multinomial regression models, increased trust in COVID-19 clinical trial information sources was associated with a 267% greater likelihood of being somewhat willing to participate compared to being unwilling and a 467% greater likelihood of being very willing. COVID-19 clinical trial awareness was associated with a 164% increased likelihood of being very willing compared to being unwilling to participate in a treatment trial. Recent challenges related to social determinants of health were not associated with willingness to participate in a COVID-19 treatment trial (Table 3).

**Table 3.** Unadjusted and adjusted multinomial regression of predictors for willingness to sign Up for a COVID-19 treatment trial or vaccine trial

Willingness to sign up for a COVID-19 treatment trial					
		Bivariate model		Multivariable model	
Outcome level^1^	Term	Unadjusted OR	P-value	^2^Adjusted OR	P-value
Somewhat willing	Challenge subscale	0.672	0.064		
	Trust in COVID-19 clinical trial info sources subscale	3.959	<0.001	3.665	0.000
	COVID-19 clinical trial awareness	1.102	0.738	1.109	0.749
Very willing	Challenge subscale	0.966	0.848		
	Trust in COVID-19 clinical trial info sources subscale	5.354	0.000	5.672	0.000
	COVID-19 clinical trial awareness	2.562	0.000	2.637	0.001
Willingness to sign up for a COVID-19 vaccine trial					
Outcome level^1^	Term	Unadjusted OR	P-value	^2^Adjusted OR	P-value
Somewhat willing	Challenge subscale	0.824	0.327		
	Trust in COVID-19 clinical trial info sources subscale	3.838	0.000	3.927	0.000
	COVID-19 clinical trial awareness	1.227	0.452	1.164	0.625
Very willing	Challenge subscale	0.837	0.371		
	Trust in COVID-19 clinical trial info sources subscale	5.368	0.000	5.259	0.000
	COVID-19 clinical trial awareness	2.592	0.000	2.407	0.004

1 Not willing is the reference group.

2 Adjusted for gender, income, and age.

In the adjusted multinomial regression models, increased trust in COVID-19 clinical trial information sources was associated with a 293% greater likelihood of being somewhat willing to participate compared to being unwilling and a 426% greater likelihood of being very willing. COVID-19 clinical trial awareness was also associated with a 141% increased likelihood of being very willing compared to being unwilling to participate in a vaccine trial. Recent challenges related to social determinants of health were not associated with a willingness to participate in a COVID-19 vaccine trial (Table 3).

### Race/ethnicity as a moderator of willingness to participate in COVID-19 clinical trials

The interaction of social determinants of health challenges with race/ethnicity was significant (*p* = .039) for willingness to participate in COVID-19 treatment trials. When analyses were stratified by race/ethnicity, having more social determinants of health challenges was associated with a slight increased likelihood of willingness to participate in a COVID-19 treatment trial for Black respondents (OR = 1.02 for those who were somewhat willing and OR = 1.26 for those who were very willing). Conversely, having more social determinants of health challenges was associated with a decreased likelihood of willingness to participate in a COVID-19 treatment trial for Latino respondents (OR = 0.541 for those who were somewhat willing and OR = 0.625 for those who were very willing).

The interaction of trust in COVID-19 trial information sources with race/ethnicity was also significant (*p* = .028) for willingness to participate in COVID-19 vaccine trials. When analyses were stratified by race/ethnicity, for Black respondents, higher trust in COVID-19 trial information sources was associated with an increased likelihood of being willing to participate among those who were somewhat willing (OR = 4.98, *p* = .001). There was a similar pattern among those who were very willing but with slightly lower odds (OR = 3.79, *p* = .002). For Latino respondents, higher trust in COVID-19 trial information sources was similarly associated with an increased likelihood of being willing to participate in a COVID-19 vaccine trial for those who were somewhat willing (OR = 4.12, *p* = < .001) and a significantly higher likelihood for those who were very willing compared to those who were unwilling (OR = 11.36, *p* = < .001). None of the interaction terms for willingness to participate in COVID-19 treatment trials were significant.

### Trust in sources of information about COVID-19 clinical trials by race/ethnicity

Additionally, participants indicated that the most trusted source of COVID-19 trial information was their doctor or healthcare provider, and the least trusted source of COVID-19 trial information was friends, family, and community leaders. There was a statistically significant difference between Black and Latino participants for trust in each information source, with Black participants generally exhibiting slightly less trust than Latino participants (Table 4).

**Table 4.** Trust in sources of information about COVID-19 clinical trials by race/ethnicity

	Total	Black participants *N* = 230	Latino participants *N* = 368	P-value
The National Institutes of Health (NIH)				< 0.001
Not at all	51 (9.3%)	25 (11.8%)	26 (7.7%)	
A little	173 (31.5%)	42 (19.9%)	131 (38.8%)	
A lot	325 (59.2%)	144 (68.2%)	181 (53.6%)	
Your doctor or healthcare provider				< 0.001
Not at all	30 (5.4%)	17 (7.8%)	13 (3.9%)	
A little	143 (25.7%)	33 (15.1%)	110 (32.6%)	
A lot	383 (68.9%)	169 (77.2%)	214 (63.5%)	
Your local healthcare clinic or hospital				< 0.001
Not at all	35 (6.3%)	20 (9.1%)	15 (4.5%)	
A little	154 (27.7%)	39 (17.7%)	115 (34.2%)	
A lot	367 (66.0%)	161 (73.2%)	206 (61.3%)	
University hospitals				<0.001
Not at all	42 (7.6%)	21 (9.6%)	21 (6.3%)	
A little	170 (30.7%)	42 (19.3%)	128 (38.2%)	
A lot	341 (61.7%)	155 (71.1%)	186 (55.5%)	
Companies that make drugs for medical use				0.002
Not at all	89 (16.3%)	45 (21.1%)	44 (13.2%)	
A little	225 (41.1%)	69 (32.4%)	156 (46.7%)	
Very much	233 (42.6%)	99 (46.5%)	134 (40.1%)	
People who do research				< 0.001
Not at all	53 (9.7%)	31 (14.5%)	22 (6.6%)	
A little	222 (40.7%)	60 (28.0%)	162 (48.8%)	
A lot	271 (49.6%)	123 (57.5%)	148 (44.6%)	
Friends, family, and community leaders				< 0.001
Not at all	63 (11.6%)	33 (15.5%)	30 (9.0%)	
A little	254 (46.6%)	53 (24.9%)	201 (60.5%)	
A lot	228 (41.8%)	127 (59.6%)	101 (30.4%)	

### Reasons for unwillingness to take part in a COVID-19 vaccine clinical trial

The Common Survey 2.0 included a question that gave respondents eight possible reasons for not wanting to participate in a COVID-19 vaccine trial. Our results showed that a statistically significantly larger percentage of Black respondents endorsed the following reasons for not wanting to participate in a COVID-19 vaccine clinical trial compared with Latino respondents: “I don’t trust researchers,” “I don’t trust the government,” “The COVID-19 vaccine may not be safe,” “I don’t understand what will happen to me,” and “Vaccines in general are bad for you.” A greater percentage of Black respondents reported having health problems that would prevent them from taking part in clinical trials compared to Latino respondents. Conversely, a greater percentage of Latino respondents were concerned about the time that participating in the clinical trial would take (“It will take me time”) compared with Black respondents. A very small percentage of both Latino and Black respondents said that they didn’t think clinical trials were important or were concerned that participation in clinical trials would cost them money (Table 5).

**Table 5.** Reasons for willingness or unwillingness to take part in a COVID-19 vaccine clinical trial

	Total	Black participants *N* = 228	Latino participants *N* = 267	P-value
Possible reasons for not taking part in a clinical trial for a COVID-19 vaccine.				
I don’t trust researchers.	139 (28.1%)	81 (35.5%)	58 (21.7%)	< 0.001
I don’t trust the government.	161 (32.5%)	100 (43.9%)	61 (22.8%)	<0.001
The COVID-19 vaccine may not be safe.	131 (26.5%)	70 (30.7%)	61 (22.8%)	0.048
I don’t believe clinical trials are important.	16 (3.2%)	10 (4.4%)	6 (2.2%)	0.180
I don’t understand what will happen to me.	200 (40.4%)	129 (56.6%)	71 (26.6%)	< 0.001
It will cost me money.	15 (3.0%)	7 (3.1%)	8 (3.0%)	0.962
It will cost me time.	74 (14.9%)	14 (6.1%)	60 (22.5%)	< 0.001
Vaccines in general are bad for you.	10 (2.0%)	8 (3.5%)	2 (0.7%)	0.050
I have health problems that prevent me from taking part in a clinical trial.	57 (11.5%)	41 (18.0%)	16 (6.0%)	< 0.001
Taking part in a clinical trial for a COVID-19 vaccine would…	Total	Black participants*N* = 230	Latino participants*N* = 370	P-value
Makes me feel like I am helping keep other people healthy.				< 0.001
Strongly disagree	66 (11.4%)	3 (1.3%)	63 (17.8%)	
Disagree	52 (9.0%)	17 (7.5%)	35 (9.9%)	
Neutral	122 (21.0%)	34 (15.0%)	88 (24.9%)	
Agree	253 (43.5%)	130 (57.3%)	123 (34.7%)	
Strongly agree	88 (15.1%)	43 (18.9%)	45 (12.7%)	
Help find a vaccine for COVID-19.				< 0.001
Strongly disagree	58 (10.1%)	4 (1.8%)	54 (15.5%)	
Disagree	65 (11.3%)	17 (7.5%)	48 (13.8%)	
Neutral	137 (23.8%)	37 (16.3%)	100 (28.7%)	
Agree	227 (39.4%)	127 (55.9%)	100 (28.7%)	
Strongly agree	89 (15.5%)	42 (18.5%)	47 (13.5%)	
Help people like me get a vaccine for COVID-19.				< 0.001
Strongly disagree	64 (11.1%)	4 (1.8%)	60 (17.0%)	
Disagree	44 (7.6%)	17 (7.5%)	27 (7.7%)	
Neutral	116 (20.0%)	34 (15.0%)	82 (23.3%)	
Agree	259 (44.7%)	127 (55.9%)	132 (37.5%)	
Strongly agree	96 (16.6%)	45 (19.8%)	51 (14.5%)	

### Reasons for willingness to take Part in a COVID-19 vaccine clinical trial

The survey also asked respondents three questions that might explain why they would be willing to participate in a COVID-19 vaccine trial. For all three reasons, there was a statistically significant difference between views expressed by Black and Latino respondents: 76% of Black respondents agreed that taking part in a COVID-19 vaccine clinical trial would “make me feel like I am helping keep other people healthy” compared with 48% of Latino respondents; 75% of Black respondents agreed that participating in a vaccine trial would “help find a vaccine for COVID-19” compared with 42% of Latino participants; and 76% of Black respondents agreed that taking part in a vaccine trial would “help people like me get a vaccine for COVID-19,” compared with 52% of Latino respondents (Table 5).

## Discussion

The results of this survey highlight the profound socioeconomic challenges of the urban Black and Latino individuals who participated in this study. However, there are important similarities and differences between the Black and Latino participants in the study worth noting. For example, while a high proportion of both Black and Latino participants reported low incomes, Latino respondents reported significantly lower levels of educational attainment and health insurance coverage than Black respondents, and Black respondents reported being unemployed and unable to work due to a disability at higher rates than Latino respondents. Despite differences between the two groups, over half of Black and Latino respondents indicated that they were unwilling to participate in clinical trials, and about one-fifth were only somewhat willing. These results are consistent with other studies showing the underrepresentation of these racial/ethnic groups in clinical trials [13–16].

This study found that trust was a significant predictor of willingness to participate in COVID-19 clinical trials for both Black and Latino respondents, which has been widely recognized as an important facilitator of trial participation by people of color [19–21]. For the Black community, in particular, medical mistrust is a consequence of historical and current experiences of racism and abuse [22–24]. A study of Black/African American, Latinx/Indigenous Latin American, and Native American/Indigenous communities found that historical, cultural, and social trauma, along with social determinants of health, were related to the fear and mistrust in public health and medical institutions that influenced attitudes about COVID-19 testing and vaccination [25]. Structural racism is also a pervasive factor that influences the perceived trustworthiness of institutions by historically marginalized communities [26].

Our findings showed that the relationship between trust and willingness to participate differed between Black and Latino adults. Specifically, Black participants exhibited slightly more distrust overall than Latino participants. Despite this difference, both Black and Latino adults considered their doctor or healthcare provider the most trusted source of clinical trial information, a finding also reported by Bastida, Craig, and Walker [27–29]. This finding is consistent with prior studies demonstrating the effectiveness of healthcare providers in recruiting underrepresented groups into clinical trials [30] and emphasizing the importance of established relationships in fostering trust in health research [31].

The finding that clinical trial awareness predicts willingness to participate in trials may seem expected; however, this result is not necessarily a foregone conclusion. Individuals can still be in favor of or against participating in clinical trials whether or not they are actually aware of clinical trials being done. A recent study of Black patients and community residents found that low awareness of COVID-19 trials was a predominant barrier to trial participation [28]. The fact that Black and Latino participants who reported being aware of clinical trials indicated a greater willingness to participate in clinical trials suggests that strategies to raise trial awareness among racial/ethnic minorities is an essential first step toward increasing representation in trial research. It is also possible that the strategies used to increase familiarity with clinical trials may increase trust. Digital research recruitment registries have been utilized to raise awareness of and interest in clinical trials. While this method has shown some success, early efforts indicate that racial/ethnic minorities still lag behind in registry enrollment rates [32–33].

While the lives of both Black and Latino participants in this study are clearly impacted by social and economic factors [34], we discovered a pronounced difference between the two groups in how those factors affect engagement with clinical trials. While challenges related to social determinants of health slightly increased willingness to participate in clinical trials for Black participants, the opposite was true for Latino respondents – the more challenges they faced, the less willing they were to participate in trials. This result is not surprising, given that Latino respondents experienced more significant challenges across all measured social determinants of health, and only 58.6% reported having health insurance. Furthermore, while safety net programs may be an accessible source of support in Black communities, restrictive policies can be barriers to accessing these programs among Latino communities, especially immigrant Latino communities with undocumented legal status [35]. For example, despite a revised public charge rule being published in September 2022 that limited federal immigration officials from considering public benefits receipt in granting US entry or adjustment to permanent resident status, there is a widespread perception among immigrants that safety net program enrollment could jeopardize these processes [36–37]. This has reduced program enrollment, and without housing, food, and healthcare safety nets, Latino communities may perceive the potentially adverse personal outcomes related to clinical trial participation as outweighing the potential benefits. Therefore, increasing confidence that participation in trials will not jeopardize already fragile living situations may be critical within Latino communities. One qualitative study showed that barriers to COVID-19 vaccination for Latinos included technological literacy, language and literacy, health insurance/healthcare costs, immigration status, and location and transportation. These are likely the same barriers that stand in the way of participation in clinical trials [38].

Likewise, our findings suggest that efforts to increase trial participation among individuals with a high burden of social determinants of health challenges should be linked to community organizations that can address those challenges. Castellon-Lopez et al [39]. reached a similar conclusion, suggesting that trial accessibility and acceptability can be enhanced by addressing the needs of low-income individuals with competing financial and social demands and concerns about continuity of follow-up medical care.

Even though Black and Latino participants were equally unwilling to participate in COVID-19 clinical trials, they provided different reasons for their unwillingness. Between a quarter of Black and a third of Latino respondents said they would not participate because they “don’t trust researchers or the government,” with slightly higher levels of mistrust among Black participants. Black participants also commonly cited “I don’t understand what will happen to me” as a reason for unwillingness. Similar results of fear and mistrust by Black individuals have been noted by others [28,40]. Yet, Latino participants indicated that the time commitment was a barrier. These findings suggest that an underlying mistrust of the research process may be more salient for Black participants, as discussed above, whereas practical barriers may be the biggest impediment for Latino respondents. These differences may also be explained by a difference in the amount of time that Black and Latino participants have to participate in a trial. For instance, a greater proportion of Latino respondents were employed than Black respondents in our sample, which may explain their concerns regarding time constraints.

This study underscores the need for continued trust building to increase racial and ethnic diversity and representation in clinical trials. Research emerging from CEAL has contributed significantly to our understanding of the need for multipronged approaches to community outreach [41] and the importance of community organizations as trusted messengers [42–43]. We also know that it is important to tailor communication strategies to specific, cultural, racial, and ethnic groups and to utilize trusted messengers to disseminate clinical trial enrollment information if we are to increase trust and reduce barriers to participation in clinical research [44–47]. Additional strategies for engaging community members in research have involved the use of bilingual recruiters [48], CHWs, community health representatives (CHRs) [49–50], and community advisory councils [51–54]. For example, one CHR-led intervention led to increased awareness and ability to enroll in COVID-19 treatment and vaccine trials, increased trust in researchers, increased understanding of the potential benefit of clinical trials to others, and a decreased perception of the costs associated with clinical trial participation [55]. With growing acknowledgment of the need to engage communities of color in clinical research, researchers have begun to develop toolboxes of best practices, which have shown that being flexible, using multiple recruitment modalities, employing a bilingual research team, and incorporating the cultural values of participants can contribute to successful recruitment [56].

Current research indicates that the best practices for engaging racially and ethnically minoritized populations in research include using culturally tailored messages, ensuring that study materials address literacy levels and language needs of participants, using a variety of communication channels, and utilizing trusted leaders, religious institutions, and community organizations in community outreach efforts [57–58]. Moreover, the UK National Institute for Health Research has published guidelines to promote the inclusiveness of groups that have been historically underserved by research, which urge researchers, funders, regulators, and study teams to design studies that are simple, flexible, and tailored to the needs of different groups and that take into account local advice about the best way to reach and engage specific communities [59]. The successful inclusion of diverse communities in clinical trials will most likely require substantial investment in community outreach coupled with authentic relationships between researchers and community members that are reciprocal and mutually beneficial [60].

The limitations of this study include the fact that it was cross-sectional and that it relied primarily on convenience sampling, which precludes the ability to determine if the results are an accurate representation of the larger population. The measures are also based on self-report, which means that we are relying on the respondents to provide an accurate representation of their attitudes, behavior, and/or circumstances. Furthermore, the views expressed by the participants in this study are limited to their willingness or unwillingness to participate in COVID-19 treatment and vaccine clinical trials, specifically in the midst of a pandemic. These intentions may or may not be generalizable to willingness to participate in clinical trials for other diseases or during other periods.

## Conclusion

This study adds to the body of evidence demonstrating that mistrust is a significant barrier to participation in clinical trials by Black and Latino individuals. To successfully increase racial and ethnic diversity and representation in clinical trials, learning how to raise awareness and increase trust in clinical trials is imperative. Researchers need to continue to take measures to build trust in racial and ethnic minority communities that have been most affected by COVID-19 through deliberate and robust community engagement efforts. This has been a central tenet of the CEAL initiative, and great strides have been made toward developing best practices for including communities in the research process.

A unique finding of this study is the discovery that Latino participants were less willing to participate in clinical trials if they reported experiencing greater social challenges. These results contribute a new perspective to the feasibility of increasing the enrollment of ethnic groups in clinical trial research when they are experiencing dire social and economic stress in an uncertain immigration climate. A variety of barriers must be overcome before we can realistically expect to see progress toward increasing the representation of diverse populations in clinical trial research. This will require a persistent and multipronged approach to address the numerous and complex challenges that prevent broader participation in medical research by people of color.
